# Supplementary material for: Three-dimensional geometric morphometric analysis of the skull of Protoceratops andrewsi supports a socio-sexual signalling role for the ceratopsian frill
Source: Proc Biol Sci. 2021 Feb 3;288(1944):20202938. doi: 10.1098/rspb.2020.2938 (PMC7893235; doi:10.1098/rspb.2020.2938)
Supplement: Supplementary files [file rspb20202938supp1.pdf]

Three-dimensional geometric morphometric analysis of the skull of Protoceratops andrewsi supports a socio-sexual signalling role for the ceratopsian frill.

A. Knapp, R.J. Knell and D.W.E. Hone.

Supplementary information

Notes on specimens used in this study and additional analyses.

All specimens included in this study are from two locations in the Gobi Desert of Mongolia, Bayan Dzak (BD) and Tugrugyin Shireh (TS; Czepinski, pers. comms.). Although precise stratigraphic and locational data are not available for the majority of these specimens, particularly those collected by the AMNH in the early 20th Century, the exposures that yield *Protoceratops andrewsi* specimens in these locations are limited in area and thickness, varying from 2 to 14 metres (Dashzeveg et al., 2005). Although no precise age is available for the two locations, they are considered to be of equal or nearly equal age because of the similarity between depositional environment and fossil fauna (Jerzykiewicz et al., 1993; Hasegawa et al., 2009; Czepinski, 2020). Furthermore, it is widely accepted that *P. andrewsi* is the only recognisable ceratopsian taxon present in this formation, because of a number of autapomorphies (paired nasals, premaxillary dentition, the absence of the accessory antorbital fenestration and a U-shaped buccal crest of the dentary Czepinski, 2019; Czepinski, 2020). To allow for possible specimen differences between the two sites we employed a multivariate analysis of variance (MANOVA) on our 3D shape data, finding no significant difference between the sites in whole-skull shape data, or individual module shape data (supplementary Table 3). We also re-ran a number of analyses, including only specimens from each location (Supplementary table 8). Although some minor differences between the two locations are apparent, probably due to the higher proportion of juveniles from Tugrugyin Shireh, they do not affect the general findings of our analysis.

Two specimens (QMBC 1293 and QMBC 1294) are casts, and location data for these specimens is not known. We are able to assign these specimens to *P. andrewsi* because of the presence of previously-mentioned diagnostic features of this taxon. Nevertheless, we re-ran all analyses with these two specimens omitted to ensure that they did not adversely affect the result in any way (Supplementary Table 8). Again, the difference in results is minimal and our conclusions were not affected.

One specimen (AMNH 6409) is missing its rostral bone. In order to include this otherwise complete specimen in our analysis we estimated the missing landmarks describing the rostral with the thin plate spline (TPS) method in the R package geomorph (Adams and Otárola-Castillo, 2013). To ensure that this method of estimating shape data did not adversely influence our results, we re-ran our analyses with this specimen omitted, and again with this and the two specimens of unknown origin omitted (Supplementary Table 8). As with other additional analyses, our results were not adversely affected by the omission of these specimens.

Analyses were re-run including the original non-retrodeformed specimen data substituted in the place of their retrodeformed counterparts. The results of these analyses differed little from our main analyses which incorporated retrodeformed specimens, and again our findings were not adversely affected (Supplementary Table 8).

Finally, analyses were re-run using only anatomical landmarks (first 28 landmarks in Supplementary Table 2 and red points in Supplementary Fig. 2) to demonstrate the robustness of our high-dimensional approach (Goswami et al., 2019). The results of these analyses are also shown in Supplementary table 2. In general agreement with the analyses of Goswami et al. (2019) modules are more difficult to estimate with far fewer landmarks because a great deal of shape information is omitted. Total shape variance is also lower, but CR analysis returned a significant modular structure ($p = 0.02$) and allometry analysis is unaffected.

List of institute abbreviations

AMNH: American Museum of Natural History, New York, USA. CMNH: Carnegie Museum of Natural History, Pittsburgh, USA. FMNH Field Museum of Natural History, Chicago, USA. MAS: Mongolian Academy of Sciences, Ulaanbaatar, Mongolia. NHMUK: The Natural History Museum, London, UK. QMBC: School of Biological and Chemical Sciences, Queen Mary University of London, UK. ZPAL: Institute of Paleobiology, Polish Academy of Sciences, Warsaw, Poland.

References

Adams DC and Otárola-Castillo E (2013). Geomorph: an R package for the collection and analysis of geometric morphometric shape data. *Methods in Ecology and Evolution.* **4;** 393 – 399.

Czepiński Ł (2019). Ontogeny and variation of a protoceratopsid dinosaur *Bagaceratops rozhdestvenskyi* from the Late Cretaceous of the Gobi Desert. *Historical Biology*. doi: 10.1080/08912963.2019.1593404.

Czepiński Ł (2019). New protoceratopsid specimens improve the age correlation of the Upper Cretaceous Gobi Desert strata. *Acta Palaeontol. Pol.* **65.** Doi: 10.4202/app.00701.2019.

Dashzeveg D, L Dingus DB, Loope CC, Dulam ST, and Sweeney MR (2005). New stratigraphic subdivision, depositional environment, and age estimate for the Upper Cretaceous Djadokhta Formation, Southern Ulan Nur Basin, Mongolia. *American Museum Novitates* **3498**:1–31.

Goswami A, Watanabe A, Felice RN, Bardua C, Fabre AC and Polly PD (2019). High-density morphometric analysis of shape and integration: the good, the bad, and the not-really-a-problem. *Integrative and Comparative Biology.* Doi: 10.1093/icb/icz120.

Hasegawa H, Tada R, Ichinnorov N, and Minjin C (2009). Lithostratigraphy and depositional environments of the Upper Cretaceous Djadokhta Formation, Ulan Nuur basin, southern Mongolia, and its paleoclimatic implication. *Journal of Asian Earth Sciences.* **35**: 13–26.

Jerzykiewicz T, Currie PJ, Eberth DA, Johnston PA, Koster EH, and Zheng JJ (1993). Djadokhta Formation correlative strata in Chinese Inner Mongolia: an overview of the stratigraphy, sedimentary geology, and paleontology and comparisons with the type locality in the pre-Altai Gobi. *Canadian Journal of Earth Sciences.* **30**: 2180–2195.

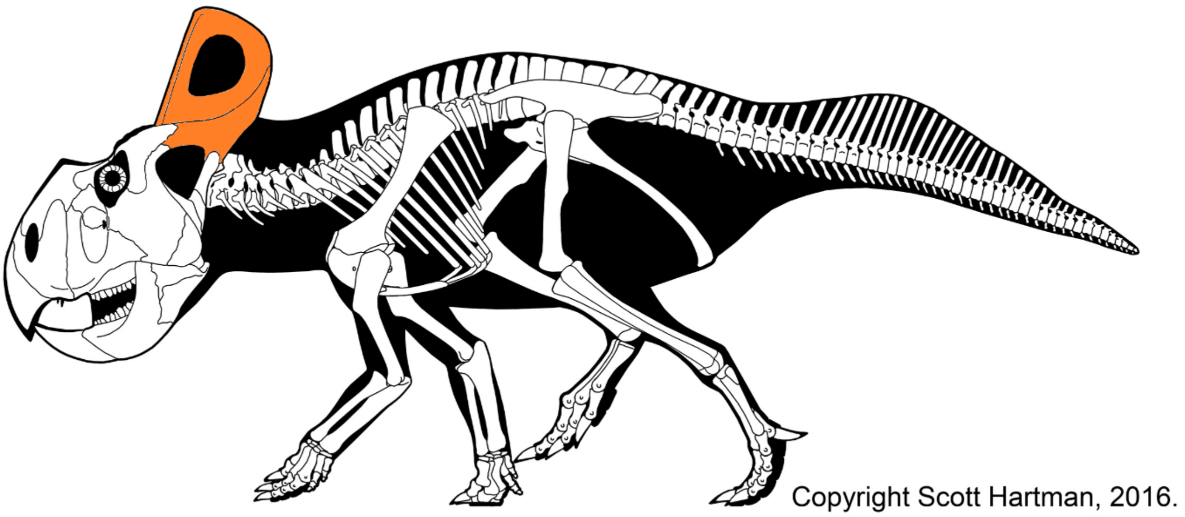

Supplementary Figure 1: Illustration of adult *Protoceratops andrewsi* skeleton, showing the prominent parietal-squamosal frill highlighted in orange.

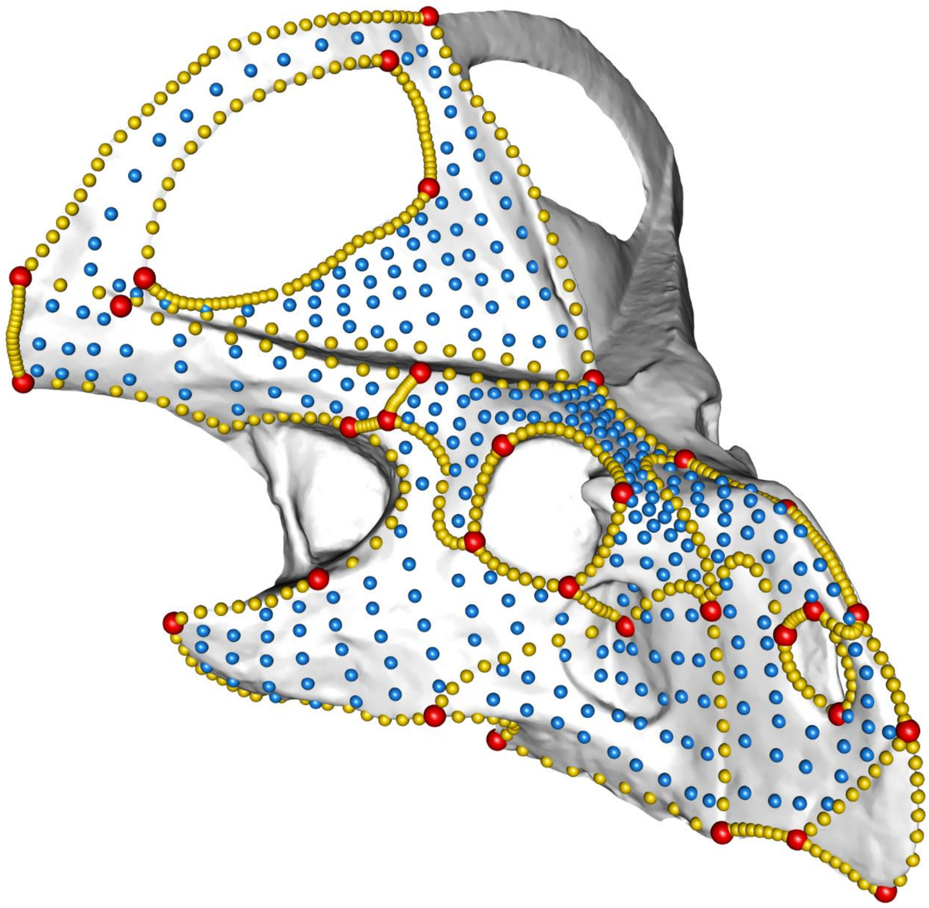

Supplementary Figure 2: Layout of landmarks on specimen of *P. andrewsi* skull. Anatomical landmarks are shown as red points, points on semilandmark curve are coloured yellow, and surface patch semilandmarks are shown in blue. Due to obliteration of sutures between lachrymal, frontal, prefrontal and postorbital in many mature specimens, these elements are treated as a single element (i.e. postorbital).

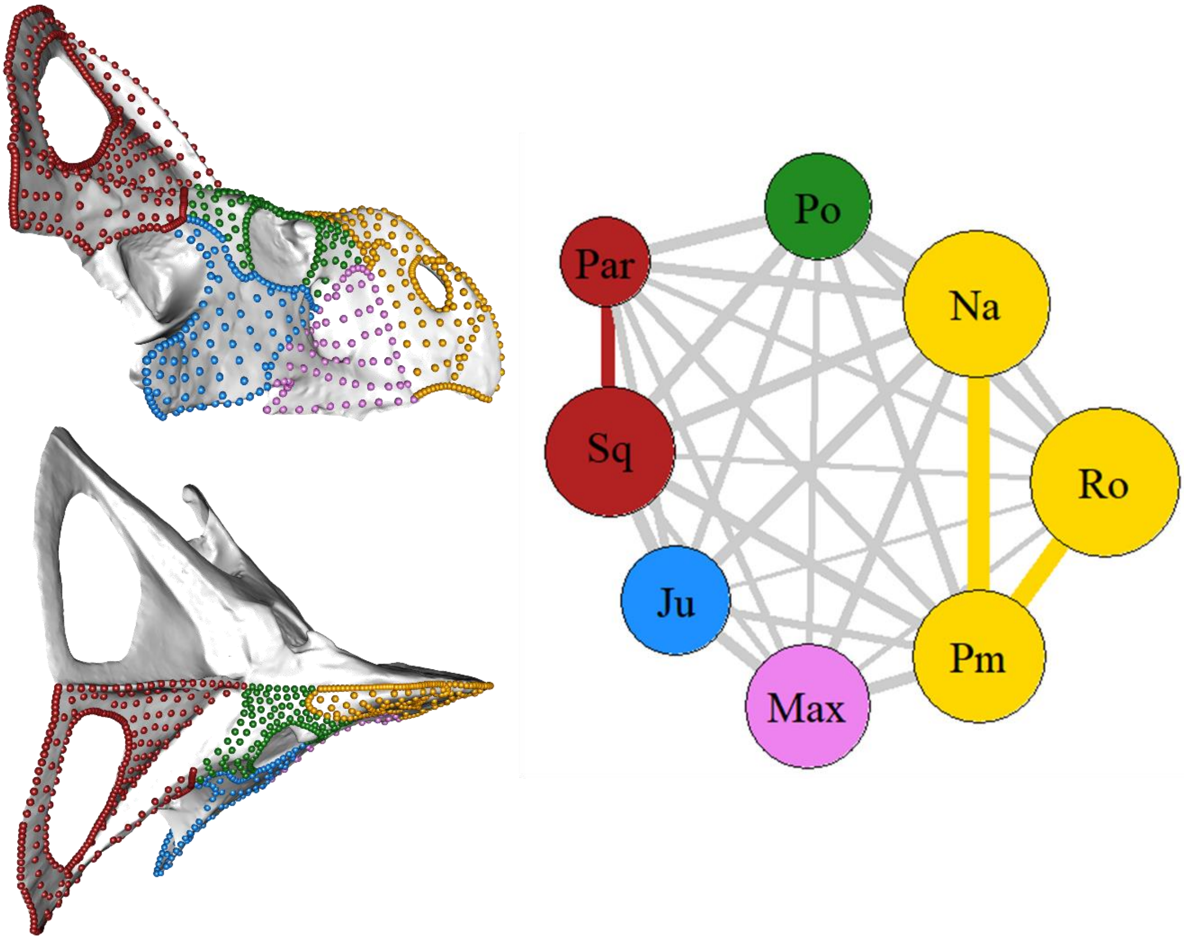

Supplementary Figure 3: Modules identified in the skull of *P. andrewsi* from *EMMLi* analysis. Meshes on the left show right lateral (left upper image) and dorsal (left lower image) views of specimen AMNH 6439, with landmarks colour-coded according to module. The network graph (right) shows all analysed skull elements, arranged corresponding to their relative position in the skull and coloured according to module. Node size is scaled according to within-module correlation value. Between-module correlations are represented by connecting lines. Line thickness represents relative correlation value, and where to modules are merged the lines are coloured appropriately. **Par:** parietal; **Sq:** squamosal; **Ju:** jugal; **Po**: postorbital; **Na:** nasal; **Max:** maxilla; **Pm:** premaxilla; **Ro:** rostral. Module colours are **frill**: red; **jugal**: blue; **postorbital**: green; **maxilla:** pink; **snout:** yellow.

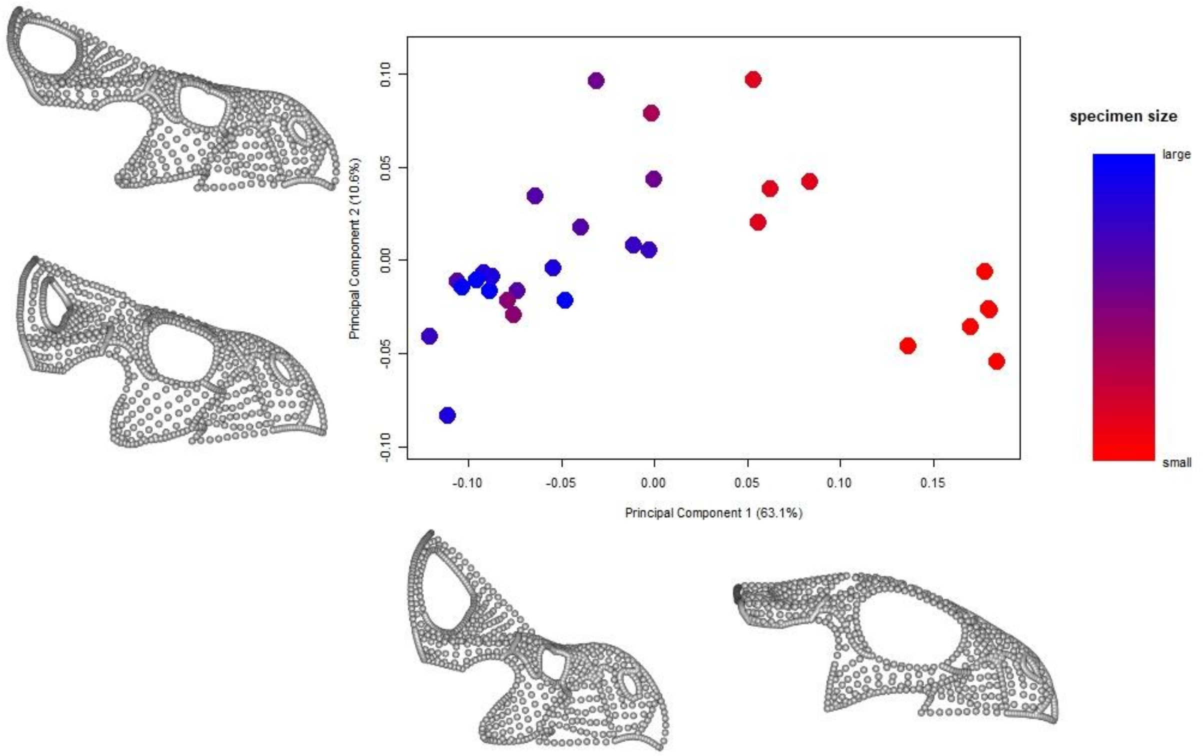

Supplementary Figure 4: Principal component analysis of 30 complete *P. andrewsi* skulls, landmarked with 821 points and including surface semilandmarks. PC1 accounts for 61.8% of total shape variation and describes a transition from a shape typical of small individuals (with short, flattened frills) at positive values to a shape typical of the largest individuals (large, upright frills) at negative values. PC2 accounts for 10.4% of total shape variation and describes a transition from a narrow, flattened, backwards-extending frill to a more upright and wider frill. The first 11 and 20 principal components account for 95% and 99% of total shape variation respectively.

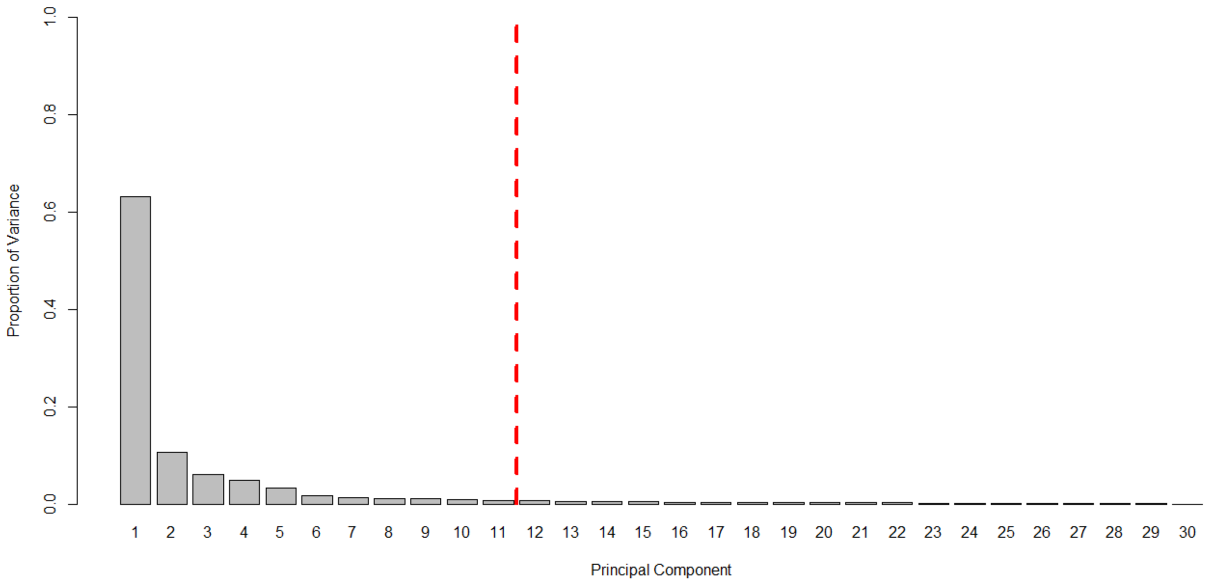

Supplementary Figure 5: Scree plot showing proportion of variance accounted for by each principal component. The red dashed line represents a cumulative variance of 95%.

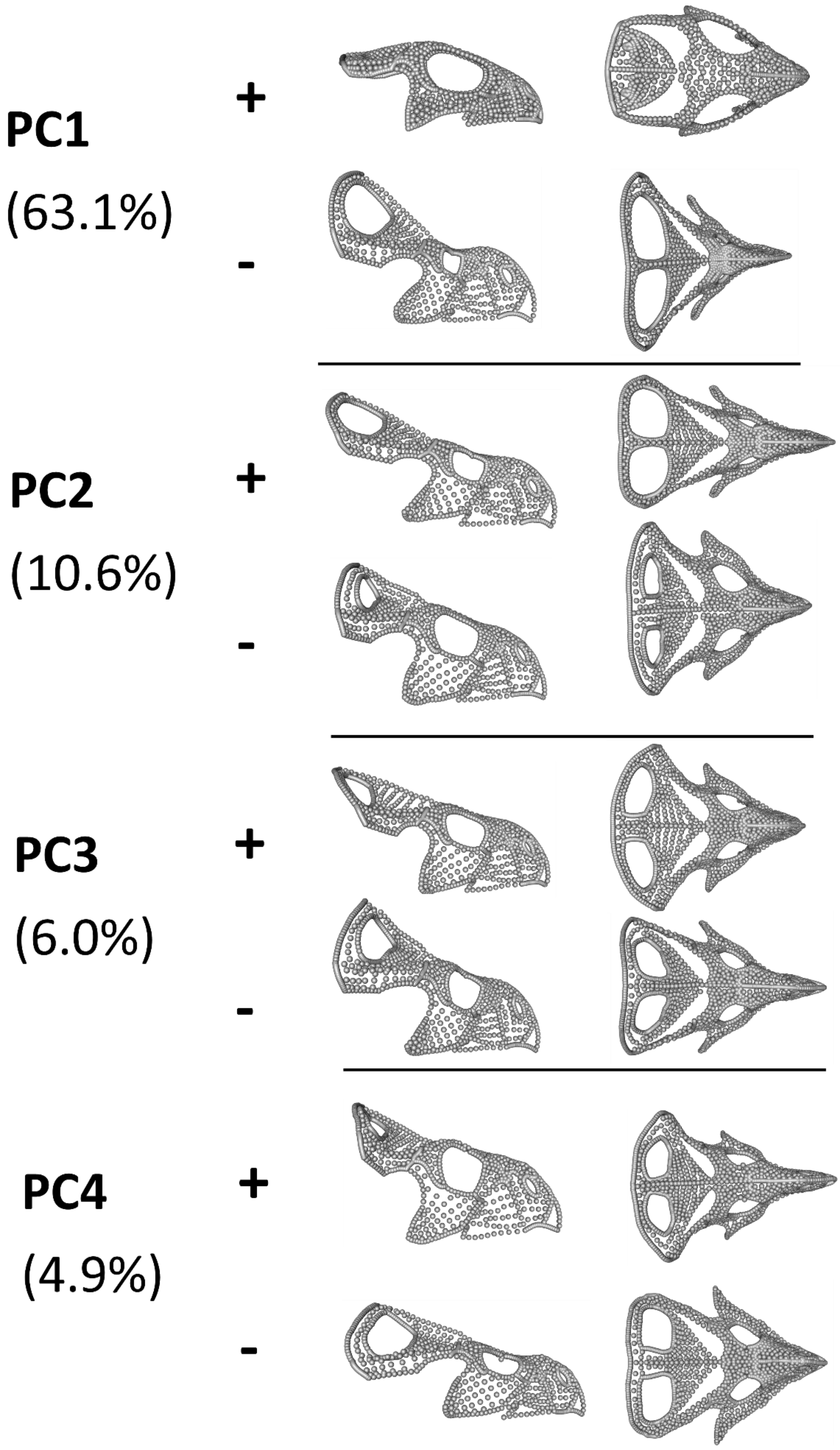

Supplementary Figure 6: Projected extreme shapes (+ and -) for first 4 principal components. Shown are left lateral (left column) and dorsal (right column) views. Dorsal views are mirrored across the midline. Percentages in parentheses represent contributions to shape variation of each PC.

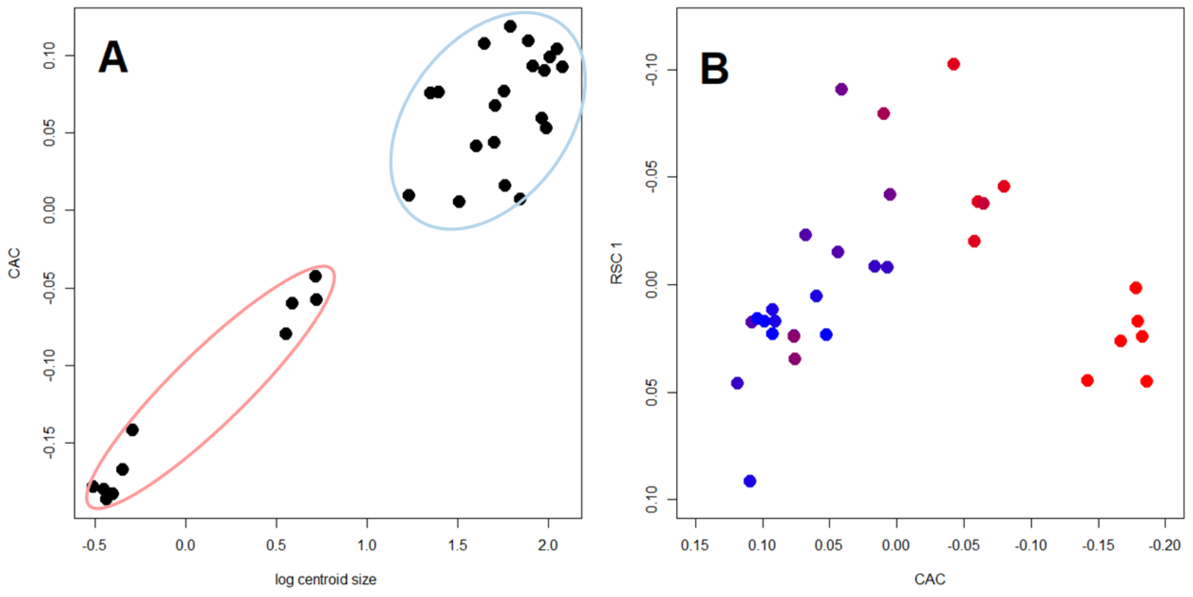

Supplementary Figure 7: Results of allometry analysis for whole-skull data. Shown are regression of common allometric component (CAC) against centroid size (Fig. 7A), and CAC against residual shape component (RSC) 1 (Fig. 7B). Fig. 7A also shows groupings of 'juvenile' (red ellipse) and 'adult' (blue ellipse) specimens, based on relative position within this plot (note that the x axis, size, is a log scale).

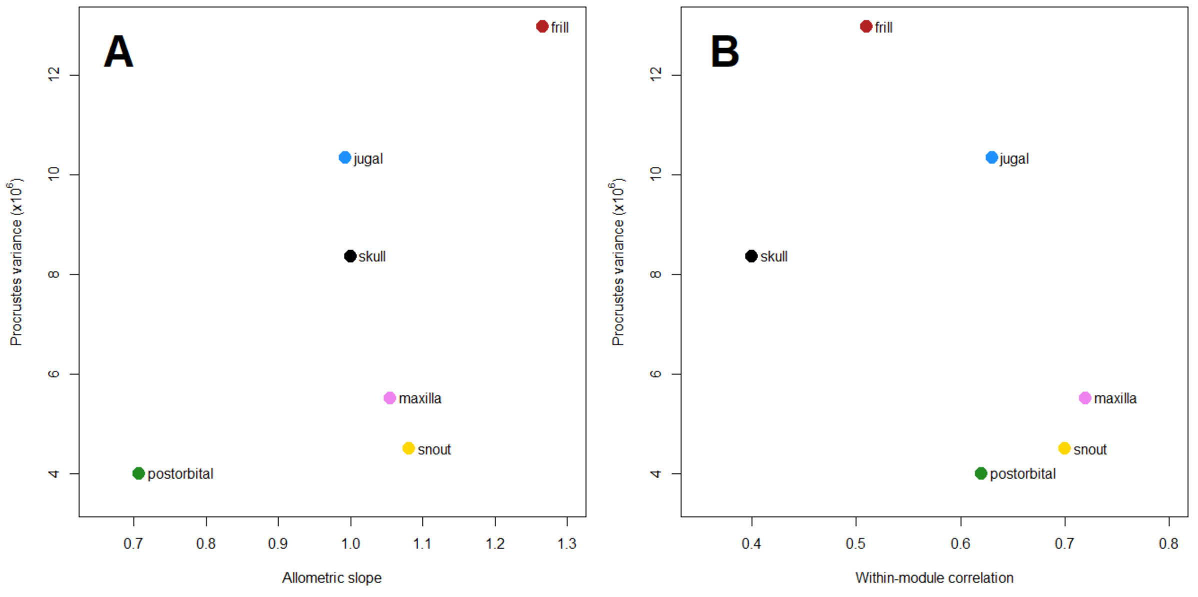

Supplementary Figure 8: Relationship between mean Procrustes variance and growth rate (Allometric slope; A) and Procrustes variance and within-module correlation (B). Neither relationship is significant. Procrustes variance in both plots is allometry-corrected and divided by number of landmarks in each module.

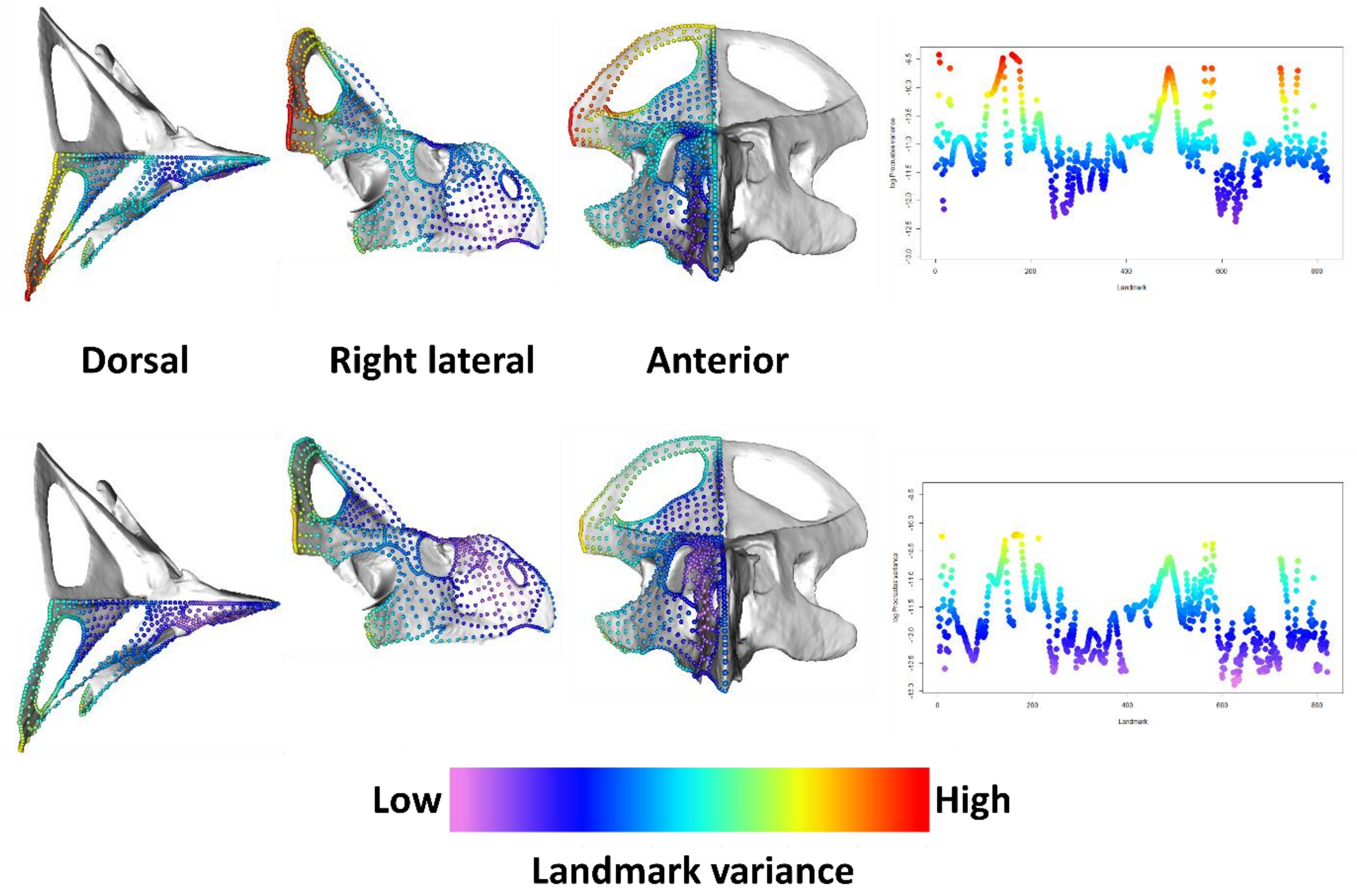

Supplementary Figure 9: Relative per-landmark variance in the skull of *P. andrewsi*, showing raw variance (top row) and variance adjusted for allometry (bottom row). Landmarks are log-transformed and are colour-coded from least variable (purple) to most variable (red).

Supplementary Table 1: List of specimens used in study. (BD: Bayan Dzak; TS: Tugrugyin Shireh)

						Inclusion in dip test analyses						
**Species**	**Institution**	**Specimen number**	**locality**	**retrodeformed**	**mirrored**	**Whole skull**	**Frill**	**Snout**	**Postorbital**	**Jugal**	**Maxilla**	**notes**
Protoceratops andrewsi	AMNH	6408	**BD**	N	Y	Y	Y	Y	Y	Y	Y	
Protoceratops andrewsi	AMNH	6409	**BD**	N	Y	Y	Y	Y	Y	Y	Y	Rostral landmarks estimated using TPS method (39)
Protoceratops andrewsi	AMNH	6413	**BD**	N	N	Y	Y	Y	Y	Y	Y	
Protoceratops andrewsi	AMNH	6414	**BD**	N	N	Y	Y	Y	Y	Y	Y	
Protoceratops andrewsi	AMNH	6419	**BD**	N	N	Y	Y	Y	Y	Y	Y	
Protoceratops andrewsi	AMNH	6425	**BD**	N	N	Y	Y	Y	Y	Y	Y	
Protoceratops andrewsi	AMNH	6429	**BD**	Y	Y	Y	Y	Y	Y	Y	Y	
Protoceratops andrewsi	AMNH	6431	**BD**	N	Y	Y	Y	Y	Y	Y	Y	
Protoceratops andrewsi	AMNH	6433	**BD**	N	N	Y	Y	Y	Y	Y	Y	
Protoceratops andrewsi	AMNH	6434	**BD**	Y	N	Y	Y	Y	Y	Y	Y	
Protoceratops andrewsi	AMNH	6439	**BD**	N	Y	Y	Y	Y	Y	Y	Y	
Protoceratops andrewsi	AMNH	6466	**BD**	Y	N	Y	Y	Y	Y	Y	Y	
Protoceratops andrewsi	AMNH	6467	**BD**	N	N	Y	Y	Y	Y	Y	Y	
Protoceratops andrewsi	CMNH	9185	**BD**	Y	N	Y	Y	Y	Y	Y	Y	
Protoceratops andrewsi	MAS	100.503	**TS**	N	Y	Y	Y	Y	Y	Y	Y	
Protoceratops andrewsi	MAS	100.517	**TS**	N	N	Y	Y	Y	Y	Y	Y	
Protoceratops andrewsi	MAS	100.519	**TS**	N	N	Y	Y	Y	Y	Y	Y	
Protoceratops andrewsi	MAS	100.523	**TS**	N	Y	Y	Y	Y	Y	Y	Y	
Protoceratops andrewsi	MAS	100.524	**TS**	N	Y	Y	Y	Y	Y	Y	Y	
Protoceratops andrewsi	MAS	100.526.C	**TS**	N	Y	Y	Y	Y	Y	Y	Y	
Protoceratops andrewsi	MAS	100.530.B	**TS**	N	Y	Y	Y	Y	Y	Y	Y	
Protoceratops andrewsi	MAS	100.530.D	**TS**	N	Y	Y	Y	Y	Y	Y	Y	
Protoceratops andrewsi	MAS	100.530.G	**TS**	N	Y	Y	Y	Y	Y	Y	Y	
Protoceratops andrewsi	MAS	100.530.H	**TS**	Y	Y	Y	Y	Y	Y	Y	Y	
Protoceratops andrewsi	MAS	100.530.K	**TS**	Y	N	Y	Y	Y	Y	Y	Y	
Protoceratops andrewsi	MAS	100.531	**TS**	N	N	Y	Y	Y	Y	Y	Y	Cast of MAS 100.531
Protoceratops andrewsi	MAS	100.534	**TS**	N	N	Y	Y	Y	Y	Y	Y	
Protoceratops andrewsi	NHMUK	5134	**BD**	N	N	Y	Y	Y	Y	Y	Y	Cast of FMNH PR 14045
Protoceratops andrewsi	QMBC	1293	**?**	N	Y	Y	Y	Y	Y	Y	Y	Cast (original unknown)
Protoceratops andrewsi	QMBC	1294	**?**	N	N	Y	Y	Y	Y	Y	Y	Cast (original unknown)
Protoceratops andrewsi	AMNH	6418	**BD**	N	N	N	Y	N	N	Y	Y	
Protoceratops andrewsi	AMNH	6438	**BD**	N	Y	N	N	Y	Y	Y	Y	
Protoceratops andrewsi	AMNH	6637	**BD**	N	N	N	N	Y	Y	Y	Y	
Protoceratops andrewsi	MAS	100.500	**TS**	N	N	N	Y	N	Y	Y	Y	
Protoceratops andrewsi	MAS	100.502	**TS**	Y	Y	N	Y	N	Y	Y	Y	
Protoceratops andrewsi	MAS	100.526.B	**TS**	Y	N	N	Y	N	Y	Y	Y	
Protoceratops andrewsi	MAS	100.537	**TS**	N	N	N	Y	N	N	N	Y	
Protoceratops andrewsi	MAS	100.539	**TS**	N	N	N	N	N	Y	Y	Y	
Protoceratops andrewsi	MAS	100.544	**TS**	N	N	N	N	N	N	Y	N	
Protoceratops andrewsi	MAS	100.550	**TS**	N	N	N	Y	N	N	N	N	
Protoceratops andrewsi	MAS	100.551	**TS**	N	Y	N	Y	N	Y	N	Y	
Protoceratops andrewsi	MAS	100.552	**TS**	N	Y	N	N	Y	Y	Y	Y	
Protoceratops andrewsi	ZPAL	MgD-II.2b	**BD**	N	N	N	N	N	N	Y	Y	
Protoceratops andrewsi	ZPAL	MgD-II.5	**BD**	N	Y	N	Y	N	N	N	N	

Supplementary Table 2: Landmark definitions for anatomical landmarks and semilandmarks curves (surface semilandmarks not included).

Landmark	Description
1	Distal tip of rostrum
2	Upper junction of rostrum and maxilla in midline
3	Junction of maxilla and nasal on midline
4	Tip of nasal horn
5	Junction of nasal and postorbitals on midline
6	Midline anterior tip of parietal
7	Rearmost point of parietal on midline
8	Junction of parietal and squamosal at outermost edge of frill
9	Lower angle of squamosal on frill margin
10	Innermost contact point of squamosal and quadrate
11	Innermost contact point of quadrate and jugal
12	Tip of jugal
13	Contact of maxilla and jugal at lower edge of zygomatic ridge
14	Junction of maxilla, jugal, and lachrymal
15	Rearmost junction of premaxilla and maxilla
16	Junction of maxilla and premaxilla at lower margin of skull
17	Rearmost point of maxilla on tooth row
18	Junction of premaxilla and rostrum on lower margin of skull
19	Junction of premaxilla and nasal on rear margin of nares at narial process
20	Midpoint of line between points s18 and s17 on margin of narial fossa
21	Midpoint of line between points s19 and s2 on margin of narial fossa
22	Junction of jugal and lachrymal on orbit margin
23	Junction of lachrymal and postorbital on orbit margin
24	Upper posterior margin of orbit
25	Junction of jugal and postorbital on orbit margin
26	Junction of jugal, squamosal and postorbital
27	Junction of squamosal and postorbital on margin of supratemporal fenestra
28	Outermost point of supratemporal fossa on squamosal
29	Anterior inner margin of parietal fenestra
30	Posterior inner margin of parietal fenestra
31	Posterior outer margin of parietal fenestra
32	Anterior outer margin of parietal fenestra

	**Semilandmark curves**
32-40	Landmarks 1-2 along rostral midline
41-48	Landmarks 2-3 along premaxilla midline
49-65	Landmarks 3-4 along nasal midline
66-82	Landmarks 4-5 along nasal midline
83-90	Landmarks 5-6 along postorbital midline
91-107	Landmarks 6-7 along parietal midline
108-142	Landmarks 7-8 along rear margin of parietal
143-159	Landmarks 8-6 along basal margin of parietal and supratemporal fossa
160-176	Landmarks 8-9 along exterior margin of squamosal to tip of squamosal angle

177-193	Landmarks 9-10 along margin of squamosal to junction of jugal and squamosal
194-201	Landmarks 10-11 around margin of infratemporal fenestra
202-209	Landmarks 11-12 along caudal margin of jugal
210-226	Landmarks 12-13 along rostral margin of jugal
227-234	Landmarks 13-14 along jugal/maxilla suture
235-242	Landmarks 14-15 along lachrymal/maxilla suture
243-250	Landmarks 15-16 along premaxilla/maxilla suture
251-258	Landmarks 16-17 along basal margin of maxilla
259-266	Landmarks 17-13 along caudal margin of maxilla
267-274	Landmarks 16-18 along ventral margin of premaxilla
275-282	Landmarks 18-2 along caudal margin of rostral
283-290	Landmarks 18-1 along ventral margin of rostral
291-298	Landmarks 15-19 along premaxilla/nasal suture
299-306	Landmarks 19-20 along caudal margin of naris
307-314	Landmarks 19-21 along caudal margin of naris
315-322	Landmarks 21-3 along maxilla/nasal suture
323-330	Landmarks 20-21 along rostral margin of naris
331-338	Landmarks 14-22 along lachrymal/jugal suture
339-346	Landmarks 22-23 along rostral orbit margin
347-363	Landmarks 23-24 along dorsal margin of orbit
364-373	Landmarks 24-25 along caudal margin of orbit
372-379	Landmarks 25-22 along ventral margin of orbit
380-396	Landmarks 5-15 along nasal/postorbital suture
397-413	Landmarks 25-26 along postorbital/jugal suture
414-421	Landmarks 26-10 along jugal/squamosal suture
422-438	Landmarks 26-27 along postorbital/squamosal suture
439-446	Landmarks 27-6 along caudal margin of postorbital
447-454	Landmarks 27-28 along caudal edge of squamosal at margin with supratemporal fossa
455-471	Landmarks 29-30 along interior margin of parietal fenestra
472-488	Landmarks 30-31 along caudal margin of parietal fenestra
489-505	Landmarks 31-32 along exterior margin of parietal fenestra
506-522	Landmarks 32-29 along rostral margin of parietal fenestra

Supplementary Table 3: Results of MANOVA comparing allometry-corrected shape data between Bayan Dzak and Tugrugyin Shiree, for whole-skull and individual modules.

	R^2	***p*-value**
Whole skull	0.04	0.334
Frill	0.03	0.536
Jugal	0.04	0.404
Maxilla	0.04	0.432
Postorbital	0.07	0.116
Snout	0.06	0.164

Supplementary Table 4: Module hypotheses guide.

Hypothesis	Number of modules	Description
1	2	Parietal-squamosal frill is separate module to rest of skull
2	3	Hypothesis 1 with jugal as separate module
3	2	Jugals separate module, rest of skull integrated
4	2	Nasals separate module, rest of skull integrated
5	3	Hypothesis 1 with nasal as separate module
6	4	Hypothesis 1 with jugal and nasal as separate modules
7	3	Jugal and nasal separate modules, rest of skull integrated
8	8	All identified bones individual modules
9	2	Hypothesis 1 with postorbitals incorporated into frill module
10	3	Hypothesis 9 with nasal as separate module
11	2	Hypothesis 1 with jugal incorporated into frill module
12	2	Hypothesis 11 with nasal incorporate into frill/jugal module
13	2	Hypothesis 1 with nasal incorporated into frill
14	2	Hypothesis 3 with nasal and jugal incorporated into single module
15	7	Hypothesis 8 with nasal and premax incorporated into single module
16	7	Hypothesis 8 with rostrum and premax incorporated into single module
17	6	Hypothesis 8 with nasal, rostrum and premax incorporated into separate module
18	7	Hypothesis 8 with parietal and squamosal incorporated into single module
19	7	Hypothesis 8 with nasal and postorbital incorporated into single module
20	7	Hypothesis 8 with squamosal and jugal incorporated into single module

Supplementary Table 5: Results of maximum likelihood correlation values (EMMLi; blue cells) and covariance ratio (CR; orange cells) analyses on initial 8-module hypothesis. Results are shown for raw shape data (upper) and allometry-corrected shape data (lower).

Raw data

	Parietal	Squamosal	Jugal	Postorbital	Nasal	Premaxilla	Maxilla	Rostral
Parietal	0.52	0.85	0.76	0.78	0.71	0.72	0.75	0.81
Squamosal	0.42	0.76	0.98	0.94	0.94	0.82	0.84	0.87
Jugal	0.29	0.42	0.63	0.94	0.93	0.84	0.86	0.87
Postorbital	0.36	0.37	0.31	0.60	0.95	0.85	0.86	0.92
Nasal	0.34	0.4	0.35	0.46	0.85	0.81	0.83	0.84
Premaxilla	0.32	0.37	0.28	0.36	0.67	0.77	0.92	0.86
Maxilla	0.24	0.24	0.38	0.24	0.34	0.38	0.73	0.83
Rostral	0.25	0.18	0.16	0.34	0.32	0.62	0.2	0.86

Allometry-corrected data

Parietal	0.47	0.78	0.43	0.61	0.51	0.38	0.54	0.47
Squamosal	0.29	0.75	0.83	0.73	0.75	0.62	0.63	0.54
Jugal	0.14	0.41	0.73	0.66	0.7	0.67	0.6	0.63
Postorbital	0.16	0.28	0.27	0.63	0.86	0.63	0.59	0.65
Nasal	0.11	0.18	0.22	0.32	0.72	0.61	0.52	0.6
Premaxilla	0.11	0.16	0.13	0.15	0.47	0.67	0.77	0.71
Maxilla	0.09	0.1	0.38	0.18	0.18	0.27	0.68	0.67
Rostral	0.11	0.12	0.07	0.22	0.22	0.56	0.21	0.87

Supplementary Table 6: Results of maximum likelihood correlation values (EMMLi; blue cells) and covariance ratio (CR; orange cells) analyses on revised 5-module hypothesis. Results are shown for raw shape data (upper) and allometry-corrected shape data (lower).

Raw data

	Frill	Jugal	Postorbital	Maxilla	Snout
Frill	0.51	0.89	0.84	0.86	0.95
Jugal	0.33	0.7	0.71	0.67	0.91
Postorbital	0.36	0.4	0.62	0.77	0.85
Maxilla	0.24	0.33	0.23	0.72	0.86
Snout	0.33	0.3	0.31	0.39	0.63

Allometry-corrected data

	Frill	Jugal	Postorbital	Maxilla	Snout
Frill	0.43	0.63	0.66	0.65	0.77
Jugal	0.25	0.72	0.67	0.71	0.65
Postorbital	0.28	0.31	0.57	0.77	0.64
Maxilla	0.12	0.41	0.2	0.7	0.58
Snout	0.17	0.16	0.23	0.19	0.56

Supplementary Table 7: Results of ANCOVA comparing growth rates of modules. Shown are comparison of regression slopes of module centroid size against skull centroid size, and CAC against skull centroid size. Results for the three steepest-scaling modules in each analysis are shown.

	**Centroid size**		**Common allometric component**	
	F	*p*	*F*	*p*
**Frill/snout**	52.175	<0.0001*	36.802	<0.0001*
**Frill/maxilla**	63.789	<0.0001*	NA	NA
**Maxilla/snout**	0.9934	0.3231	NA	NA
**Postorbital/snout**	NA	NA	0.4741	0.494
**Frill/postorbital**	NA	NA	48.123	<0.0001*

Supplementary Table 8: Results of Hartigan's dip test. Shown are *p*-values for dip test performed on the first 8 residual shape components (RSC) of size-corrected shape data for whole-skull and individual modules, for all specimens and with juveniles excluded.

All specimens

**Module**	***n***	**RSC1**	**RSC2**	**RSC3**	**RSC4**	**RSC5**	**RSC6**	**RSC7**	**RSC8**
Skull	30	0.99	0.85	0.66	0.79	0.94	0.09	0.59	0.4
Frill	39	0.51	0.81	0.92	0.99	0.36	0.43	0.84	0.7
Jugal	43	0.52	0.99	0.97	0.71	0.3	0.82	0.83	0.81
Snout	33	0.2	0.8	0.6	0.67	0.2	0.7	0.65	0.82
Maxilla	41	0.71	0.95	0.69	0.98	0.98	0.32	0.87	0.92
Postorbital	39	0.99	0.60	0.6	0.87	0.62	0.22	0.89	0.8

Juveniles excluded

**Module**	***n***	**RSC1**	**RSC2**	**RSC3**	**RSC4**	**RSC5**	**RSC6**	**RSC7**	**RSC8**
Skull	20	0.5	0.64	0.91	0.58	0.63	0.83	0.81	0.59
Frill	27	0.9	0.95	0.48	0.4	0.49	0.99	0.89	0.37
Jugal	30	0.77	0.8	0.6	0.68	0.75	0.83	0.99	0.95
Snout	22	0.18	0.38	0.15	0.42	0.94	0.63	0.99	0.69
Maxilla	28	0.93	0.58	0.99	0.77	0.35	0.93	0.81	0.71
Postorbital	25	0.96	0.97	0.61	0.88	0.33	0.47	0.98	0.99

Supplementary Table 9: Summary of additional analyses performed on subsets of the whole-skull shape dataset, including subsample of landmarks (anatomical landmarks only). See Supplementary Table 1 for details of specimens.

		**EMMLi**	**Covariance Ratio**		**Allometry**		**per-landmark variance (log)**		**allometry-adjusted Procrustes variance (x 10^-6)**					
	n specimens	Modules	CR	*p*- value	R squared	*p*- value	high	low	whole skull	frill	snout	jugal	maxilla	postorbital
Full dataset, retrodeformed specimens	30	5	0.86	<0.001	0.52	0.004	-9.418	-12.478	9.04	13.98	4.73	11.32	6.15	4.25
Full dataset, raw shape data	30	5	0.86	<0.001	0.52	0.004	-9.418	-12.478	9.05	13.98	4.73	11.32	6.15	4.25
Unknown specimens excluded	28	5	0.86	<0.001	0.54	0.004	-9.469	-12.354	8.73	13.47	4.35	11.23	5.90	4.22
AMNH 6409 (estimated landmarks) excluded	29	5	0.86	<0.001	0.52	0.004	-9.418	-12.437	9.06	14.15	4.73	11.32	5.83	4.19
Unknown specimens and AMNH 6409 excluded	27	5	0.85	<0.001	0.54	0.004	-9.475	-12.423	8.64	13.15	4.29	11.48	5.91	4.25
Juveniles excluded	19	5	0.74	<0.001	0.1	0.072	-9.86	-12.982	7.7	13.85	4.33	7.38	3.63	1.95
Bayan Dzak specimens only	15	5	0.82	<0.001	0.42	0.004	-9.91	-12.95	5.79	10.05	2.63	6.38	3.29	2.07
Tugrugyin Shireh specimens only	13	5	0.87	<0.001	0.62	0.004	-9.157	-12.644	8.42	11.38	5.36	12.23	7.69	3.85
Anatomical landmarks only (n=28)	30	<5	1.1	0.016	0.51	0.004	-5.615	-7.995	0.01	NA	NA	NA	NA	NA

Additional files

Protoceratops_frill_landmarks.csv: landmark coordinate data for all frill module shape data (CSV file).

Protoceratops_jugal_landmarks.csv: landmark coordinate data for all jugal module shape data (CSV file).

Protoceratops_maxilla_landmarks.csv: landmark coordinate data for all maxilla module shape data (CSV file).

Protoceratops_postorbital_landmarks.csv: landmark coordinate data for all postorbital module shape data (CSV file).

Protoceratops_snout_landmarks.csv: landmark coordinate data for all snout module shape data (CSV file).

Protoceratops_whole_skull_raw_landmarks.csv: landmark coordinate data for unretrodeformed whole-skull shape data (CSV file).

Protoceratops_whole_skull_retrodeformed_landmarks.csv: landmark coordinate data for retrodeformed whole-skull shape data (CSV file).
